# GiniClust2: a cluster-aware, weighted ensemble clustering method for cell-type detection

**DOI:** 10.1186/s13059-018-1431-3

**Published:** 2018-05-10

**Authors:** Daphne Tsoucas, Guo-Cheng Yuan

**Affiliations:** 1 Department of Biostatistics and Computational Biology, Dana-Farber Cancer Institute, Boston, MA 02115 USA; 2 Department of Biostatistics, Harvard T.H. Chan School of Public Health, Boston, MA 02115 USA

**Keywords:** Clustering, Consensus clustering, Ensemble clustering, Single-cell, scRNA-seq, Gini index, Rare cell type

## Abstract

Single-cell analysis is a powerful tool for dissecting the cellular composition within a tissue or organ. However, it remains difficult to detect rare and common cell types at the same time. Here, we present a new computational method, GiniClust2, to overcome this challenge. GiniClust2 combines the strengths of two complementary approaches, using the Gini index and Fano factor, respectively, through a cluster-aware, weighted ensemble clustering technique. GiniClust2 successfully identifies both common and rare cell types in diverse datasets, outperforming existing methods. GiniClust2 is scalable to large datasets.

**Electronic supplementary material:**

The online version of this article (10.1186/s13059-018-1431-3) contains supplementary material, which is available to authorized users.

## Background

Genome-wide transcriptomic profiling has served as a paradigm for the systematic characterization of molecular signatures associated with biological functions and disease-related alterations, but traditionally this could only be done using bulk samples that often contain significant cellular heterogeneity. The recent development of single-cell technologies has enabled biologists to dissect cellular heterogeneity within a cell population. Such efforts have led to an increased understanding of cell-type composition, lineage relationships, and mechanisms underlying cell-fate transitions. As the throughput of single-cell technology increases dramatically, it has become feasible not only to characterize major cell types, but also to detect cells that are present at low frequencies, including those that are known to play an important role in development and disease, such as stem and progenitor cells, cancer-initiating cells, and drug-resistant cells [1, 2].

On the other hand, it remains a computational challenge to fully dissect the cellular heterogeneity within a large cell population. Despite the intensive effort in method development [3–8], significant limitations remain. Most methods are effective only for detecting common cell populations, but are not sensitive enough to detect rare cells. A number of methods have been developed to specifically detect rare cells [9–12], but the features used in these methods are distinct from those distinguishing major populations. Existing methods cannot satisfactorily detect both large and rare cell populations. A naïve approach combining features that are either associated with common or rare cell populations fails to characterize either type correctly, as a mixed feature space will dilute both common and rare cell type-specific biological signals, an unsatisfactory compromise.

To overcome this challenge, we have developed a new method, GiniClust2, to integrate information from complementary clustering methods using a novel ensemble approach. Instead of averaging results from individual clustering methods, as is traditionally done, GiniClust2 selectively weighs the outcomes of each model to maximize the methods’ respective strengths. We show that this cluster-aware weighted ensemble approach can accurately identify both common and rare cell types and is scalable to large datasets.

## Results

### Overview of the GiniClust2 method

**Fig. 1:**
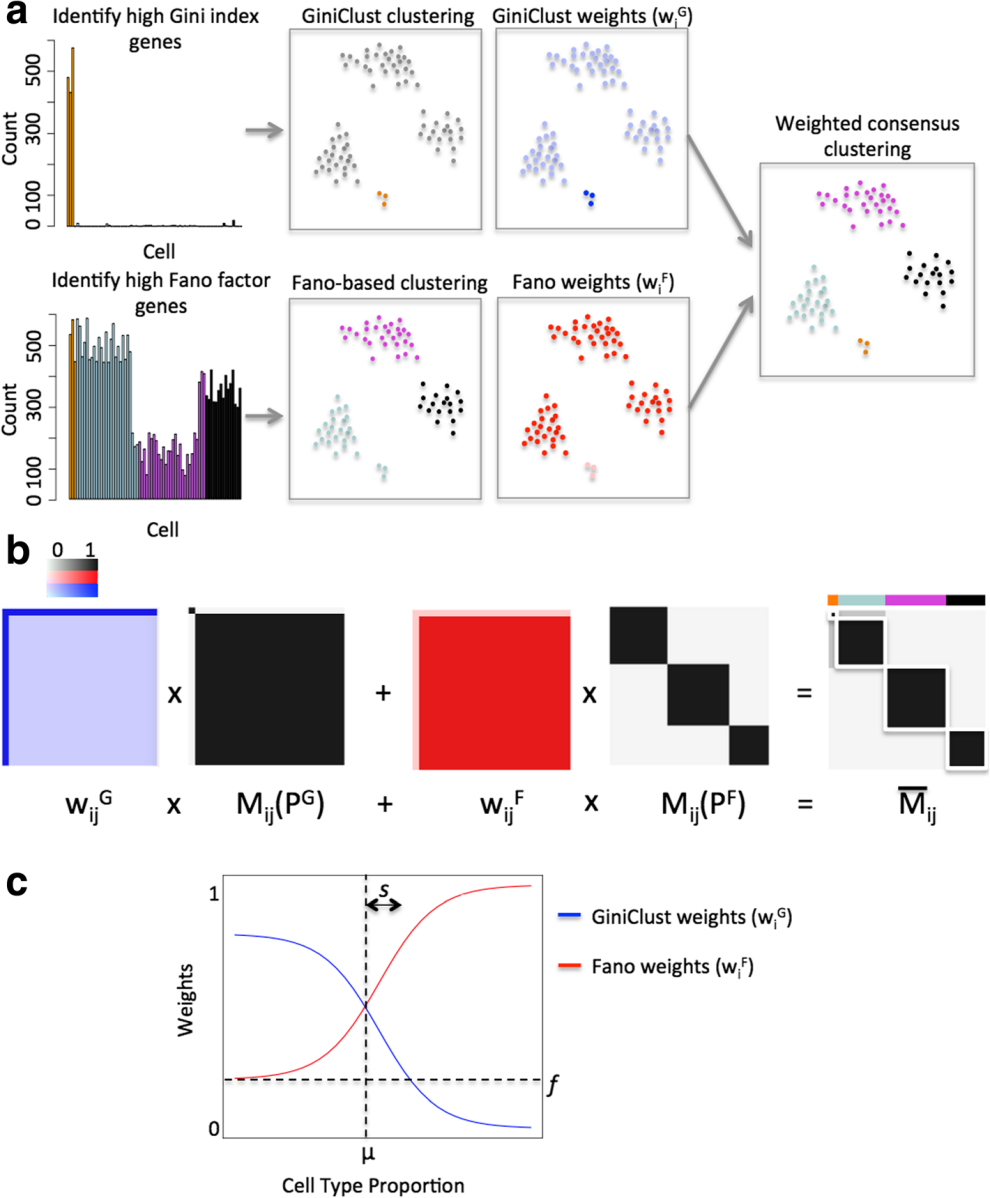
An overview of the GiniClust2 pipeline. **a** The Gini index and Fano factor are used (*left*), respectively, to select genes for GiniClust and Fano-based clustering (*middle left*). A cluster-aware, weighted ensemble method is applied to each of these, where cell-specific cluster-aware weights ${w}_i^F$ and ${w}_i^G$ are represented by the shading of the cells (*middle right*), to reach a consensus clustering (*right*). **b** A schematic of the weighted consensus association calculation, with association matrices in *black* and *white*, weighting schemes in *red* and *blue*, and final GiniClust2 clusters highlighted in *white*. **c** Cell-specific GiniClust and Fano-based weights are defined as a function of cell-type proportion, where parameters *μ*, *s*, and *f* define the shapes of the weighting curves

An overview of the GiniClust2 pipeline is shown in Fig. 1. We begin by independently running both a rare cell type-detection method and a common cell type-detection method on the same data set (Fig. 1a). In a previous study [11], we showed that different strategies are optimal for identifying genes associated with rare cell types compared to common ones. Whereas the Fano factor is a valuable metric for capturing differentially expressed genes specific to common cell types, the Gini index is much more effective for identifying genes that are associated with rare cells [11]. Therefore, we were motivated to develop a new method that combines the strengths of these two approaches. To facilitate a concrete discussion, here we choose GiniClust as the Gini index-based method and k-means as the Fano factor-based method. However, the same approach can be used to combine any other clustering methods with similar properties. We call this new method GiniClust2.

Our goal is to consolidate these two differing clustering results into one consensus grouping. The output from each initial clustering method can be represented as a binary-valued connectivity matrix, M_ij_, where a value of 1 indicates cells i and j belong to the same cluster (Fig. 1b). Given each method’s distinct feature space, we find that GiniClust and Fano factor-based k-means tend to emphasize the accurate clustering of rare and common cell types, respectively, at the expense of their complements. To optimally combine these methods, a consensus matrix is calculated as a cluster-aware, weighted sum of the connectivity matrices, using a variant of the weighted consensus clustering algorithm developed by Li and Ding [13] (Fig. 1b). Since GiniClust is more accurate for detecting rare clusters, its outcome is more highly weighted for rare cluster assignments, while Fano factor-based k-means is more accurate for detecting common clusters and therefore its outcome is more highly weighted for common cluster assignments. Accordingly, weights are assigned to each cell as a function of the size of the cluster to which the cell belongs (Fig. 1c). For simplicity, the weighting functions are modeled as logistic functions which can be specified by three tunable parameters: *μ* is the cluster size at which GiniClust and Fano factor-based clustering methods have the same detection precision, *s* represents how quickly GiniClust loses its ability to detect rare cell types, and *f* represents the importance of the Fano cluster membership in determining the larger context of the membership of each cell. The values of parameters *μ* and *s* are specified as a function of the smallest cluster size detectable by GiniClust and the parameter *f* is set to a constant (“Methods”, Additional file 1). The resulting cell-specific weights are transformed into cell pair-specific weights ${w}_{ij}^G$ and ${w}_{ij}^F$ (“Methods”), and multiplied by their respective connectivity matrices to form the resulting consensus matrix (Fig. 1b). An additional round of clustering is then applied to the consensus matrix to identify both common and rare cell clusters. The mathematical details are described in the “Methods” section.

### Accurate detection of both common and rare cell types in a simulated dataset

**Fig. 2:**
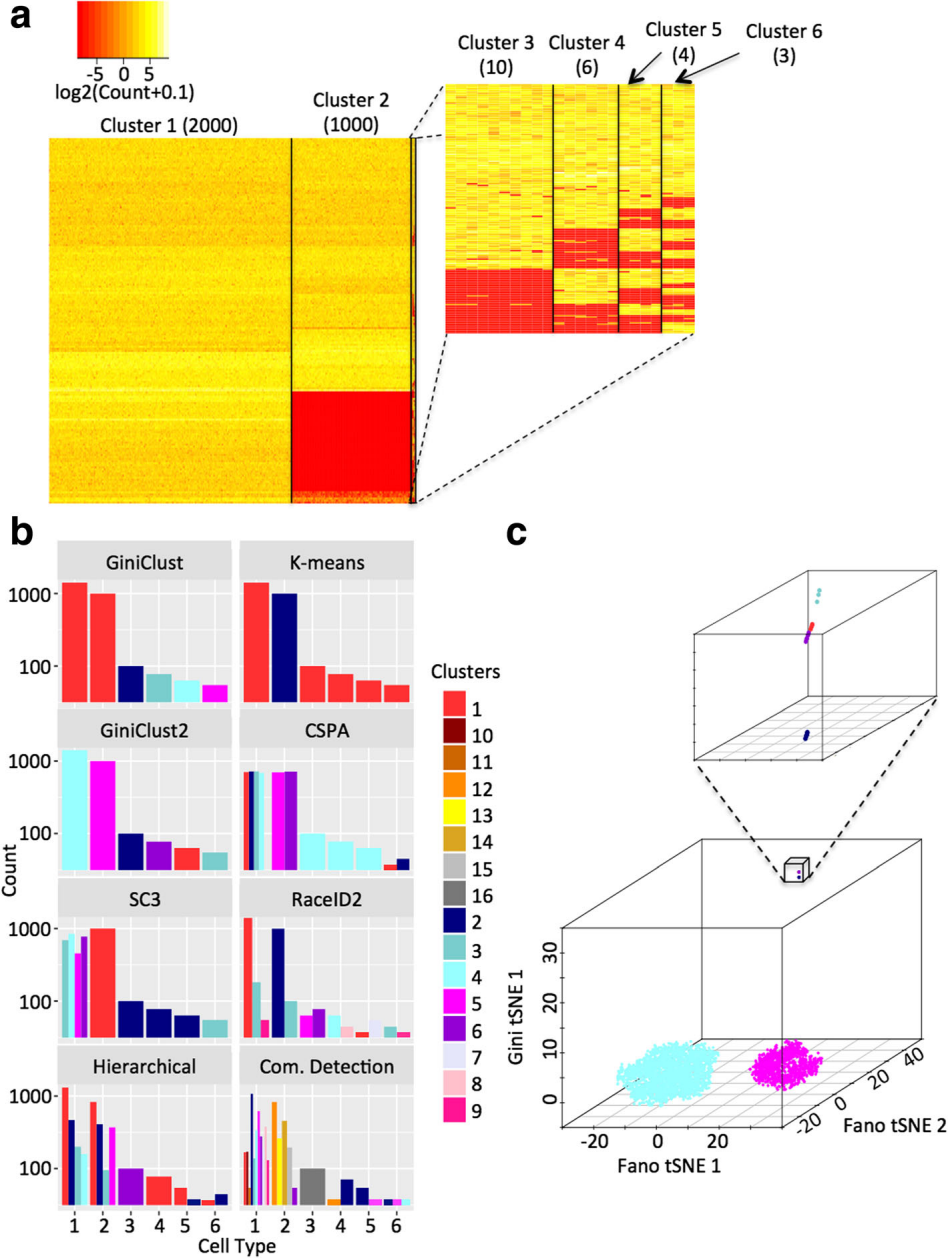
The application of GiniClust2 and comparable methods to simulated data. **a** A heatmap representation of the simulated data with six distinct clusters, showing the genes permuted to define each cluster. A zoomed-in view of the rare clusters is shown in the smaller heatmap. **b** A comparison between the true clusters (*x-axis*) and clustering results from GiniClust2 and comparable methods (*y-axis*). Each cluster is represented by a distinct *color bar*. Multiple bars are shown if a true cluster is split into multiple clusters by a clustering method. **c** A three-dimensional visualization of the GiniClust2 clustering results using a composite tSNE plot, combining two Fano-based tSNE dimensions and one Gini-based tSNE dimension. The inset shows a zoomed-in view of the corresponding region

We started by evaluating the performance of GiniClust2 using a simulated scRNA-seq dataset, which contains two common clusters (of 2000 and 1000 cells, respectively) and four rare clusters (of ten, six, four, and three cells, respectively) (“Methods”**,** Fig. 2a). We first applied GiniClust and Fano factor-based k-means independently to cluster the cells. As expected, GiniClust correctly identifies all four rare cell clusters, but merges the two common clusters into a single large cluster (Fig. 2b, Additional file 1**,** Additional file 2: Figure S1). In contrast, Fano factor-based k-means (with k = 2) accurately separates the two common clusters, while lumping together all four rare cell clusters into the largest group (Fig. 2b, Additional file 1, Additional file 2: Figure S1). Increasing k past k = 3 results in dividing each common cluster into smaller clusters, without resolving all rare clusters, indicating an intrinsic limitation of selecting gene features using the Fano factor (Additional file 2: Figure S2a). We find this limitation to be independent of the clustering method used, as applying alternative clustering methods to the Fano factor-based feature space, such as hierarchical clustering and community detection on a kNN graph, also results in the inability to resolve rare clusters (Fig. 2b, Additional file 1, Additional file 2: Figure S1). Furthermore, simply combining the Gini and Fano feature space fails to provide a more satisfactory solution (Additional file 1, Additional file 2: Figure S3). These analyses signify the importance of feature selection in a context-specific manner.

We next used the GiniClust2 weighted ensemble step to combine the results from GiniClust and Fano factor-based k-means. Of note, all six cell clusters are perfectly recapitulated by GiniClust2 (Fig. 2b, Additional file 1, Additional file 2: Figure S1), suggesting that GiniClust2 is indeed effective for detecting both common and rare cell clusters. To aid visualization, we created a composite tSNE plot, projecting the cells into a three-dimensional space based on a combination of a two-dimensional Fano-based tSNE map and a one-dimensional Gini-based tSNE map (Fig. 2c). A three-dimensional space is required because, although the Fano-based dimensions are able to clearly separate the two common clusters, the rare clusters are overlapping and cannot be fully discerned. The third (Gini) dimension results in complete separation of the rare clusters. Unlike a traditional tSNE plot, this composite view does not correspond to a single projection of a high-dimensional dataset into a three-dimensional space but integrates two orthogonal views obtained from different high-dimensional features. Although the distance does not have a simple interpretation, it provides a convenient way to visualize data from complementary views.

Since the number of common clusters is unknown in advance, we also tested the robustness of GiniClust2 with respect to other choices of k. We found that setting k = 3 provides the same final clustering, while further increase results in poorer performance by splitting of the larger clusters (Additional file 2: Figure S2b). By default, the value of k was chosen using the gap statistic, which coincided with the number of common clusters (k = 2) [14]. However, this metric may not be optimal in various cases when the underlying distribution is more complex [15]; therefore, additional exploration is often needed to select the optimal value for k. Since the clustering outcome is sensitive to the choice of k (Additional file 1), we recommend using the gap statistic as a starting point for choosing k, and then evaluating this choice of k by checking the resulting clusters for adequate separation in the Fano factor-based tSNE plot and expression of distinct and biologically relevant genes.

For comparison, we evaluated the performance of two unweighted ensemble clustering methods. First, we used the cluster-based similarity partitioning algorithm (CSPA) [16] to combine the GiniClust and Fano factor-based k-means (k = 2) clustering results. The consensus clustering splits the common clusters into six subgroups, whereas cells in the four rare clusters are assigned to one of two clusters shared with the largest common cell group (Fig. 2b, Additional file 1, Additional file 2: Figure S1). Without guidance, the consensus clustering treats all clustering results equally and attempts to resolve any inconsistency via suboptimal compromise. The second method we considered, known as SC3 [4], is specifically designed for single-cell analysis. This method performs an unweighted ensemble of k-means clusterings for various parameter choices without specifically targeting rare cell detection. Regardless of the specific parameter choices, k-means cannot resolve the rarest clusters, and the final ensemble clustering splits the largest group into three and differentiates only one of the four rare clusters (Fig. 2b, Additional file 1, Additional file 2: Figure S1). These analyses suggest that our cluster-aware, weighted ensemble approach is important for optimally combining the strengths of different methods.

We also compared the performance of GiniClust2 with other rare cell type-detection methods. In particular, we compared with RaceID2 [10], which is an improved version of RaceID [9] developed by the same group. For fair comparison, we considered k = 2, the exact number of common cell clusters, and k = 12, the parameter value recommended by authors Grün et al. as determined by a within-cluster dispersion saturation metric [10]. In both cases, RaceID2 over-estimated the number of clusters, and split both common and rare cell clusters into smaller subclusters (Fig. 2b, Additional file 1, Additional file 2: Figure S1). This tendency of over-clustering is consistent with our previous observations [11].

### Robust identification of rare cell types over a wide range of proportions

**Fig. 3:**
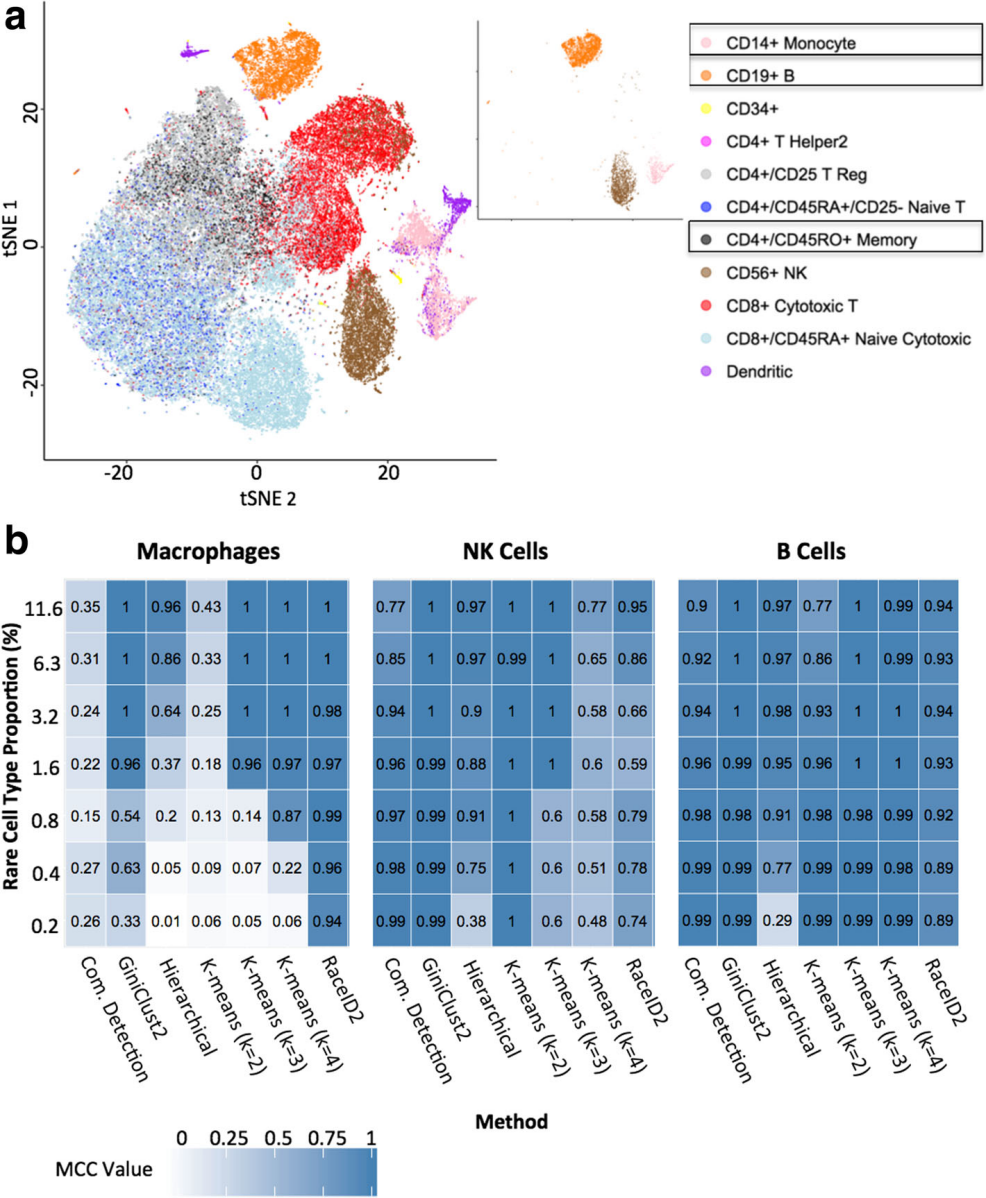
Analysis of the 68 k PBMC dataset [17]. **a** A visualization of reference labels for the full data set (*left*), along with the three cell subtypes selected for detailed analysis (*right*). **b** Comparison of the performance of different clustering methods, quantified by a Matthews correlation coefficient (MCC) [18] for each of the three cell subtypes

In order to evaluate the performance of GiniClust2 on analyzing real scRNA-seq datasets, we focused on one of the largest public scRNA-seq datasets generated by 10X Genomics [17]. The dataset consists of transcriptomic profiles of about 68,000 peripheral blood mononuclear cells (PBMCs) [17], which were classified into 11 subpopulations based on transcriptomic similarity with purified cell types (Fig. 3a). It was noted that the transcriptomic profiles of several subpopulations are nearly indistinguishable [17].

To reduce the effects of stochastic variation and technical artifacts, we started by considering only a subset of cell types whose transcriptomic profiles are distinct from one another. In particular, we focused on three large subpopulations: CD56+ natural killer (NK) cells, CD14+ monocytes, and CD19+ B cells. To ensure our analysis is not affected by within-cell type heterogeneity, additional known gene markers were used to further remove heterogeneity within each subpopulation (see “Methods” for cell type definition details). In the end, three populations were selected, corresponding to NK, macrophage, and B cells, respectively (Fig. 3a). To systematically compare the ability of different methods in detecting both common and rare cell types, we created a total of 140 random subsamples that mix different cell types at various proportions (Additional file 2: Table S1), with the rare cell type (macrophage) proportions ranging from 0.2% to 11.6% (see “Methods” for details).

We applied GiniClust2 and comparable methods to the down-sampled datasets generated above. Each method was evaluated based on its ability to detect each cell type using the Matthews correlation coefficient (MCC) [18] (Fig. 3b). The MCC is a metric that quantifies the overall agreement between two binary classifications, taking into account both true and false positives and negatives. The MCC value ranges from − 1 to 1, where 1 means a perfect agreement between a clustering and the reference, 0 means the clustering is as good as a random guess, and − 1 means a total disagreement between a clustering and the reference. In addition, we also evaluated the performance of each method using several additional metrics (Additional file 1). While each metric typically generates a different value, the relative performance across different clustering methods is highly conserved (Additional file 2: Figure S4).

RaceID2 is the best method for detecting the rare macrophage cell type at a frequency of 1.6% or lower, and GiniClust2 is the next best method. As expected, the performance of GiniClust degrades as the “rare” cell type becomes more abundant, whereas Fano factor-based k-means becomes more powerful in such cases. Combining these two methods enables GiniClust2 to perform among the top over a wide range of rare cell proportions. The remaining methods cannot detect rare cell clusters well. For the common groups, Fano factor-based k-means tends to perform better, but only if the parameter is chosen correctly. For example, Fano factor-based k-means with k = 4 systematically splits the largest NK cell group and leads to a relatively low MCC value. Other clustering methods that use Fano factor-based feature selection (such as hierarchical clustering and community detection) also adequately pick up common clusters. This strong performance is preserved by the GiniClust2 method. In comparison, RaceID2 does not perform as well here, since some of the cells in the common groups are falsely identified as rare cells. Taken together, these comparative results suggest that GiniClust2 reaches a good balance for detecting both common and rare clusters. The same conclusion can be arrived at using alternative evaluation metrics (Additional file 2: Figure S4).

### Detection of rare cell types in differentiating mouse embryonic stem cells

To test if GiniClust2 is useful for detecting previously unknown, biologically relevant cell types, we analyzed a published dataset associated with leukemia inhibitory factor (LIF) withdrawal-induced mouse embryonic stem cell (mESC) differentiation [19]. Previously, we applied GiniClust to analyze a subset containing undifferentiated mESCs, and identified a rare group of Zscan4-enriched cells [11]. As expected, these rare cells were rediscovered using GiniClust2.

In this study, we focused on the cells assayed on day 4 post-LIF withdrawal, and tested if GiniClust2 might uncover greater cellular heterogeneity than previously recognized. GiniClust2 identified two rare clusters consisting of five and four cells, respectively, corresponding to 1.80% and 1.44% of the entire cell population. The first group contains 25 differentially expressed genes when compared to the rest of the cell population (MAST likelihood ratio test *p* value < 1e-5, fold change > 2), including known primitive endoderm (PrEn) markers such as *Col4a1*, *Col4a2*, *Lama1*, *Lama2*, and *Ctsl*. These genes are also associated with high Gini index values. Overall there is a highly significant overlap between differentially expressed and high Gini genes (Fisher exact test *p* value < 1e-18). The second group contains ten differentially expressed genes (MAST likelihood ratio test *p* value < 1e-5, fold change > 2), including maternally imprinted genes *Rhox6*, *Rhox9*, and *Sct*, all of which are also high Gini genes. Once again there is a significant overlap between differentially expressed and high Gini genes (Fisher exact test *p* value < 1e-12). Although these clusters were detected in the original publication [19], this was achieved based on a priori knowledge of relevant markers. Here, the strength of GiniClust2 is that it can identify these clusters without previous knowledge.

**Fig. 4:**
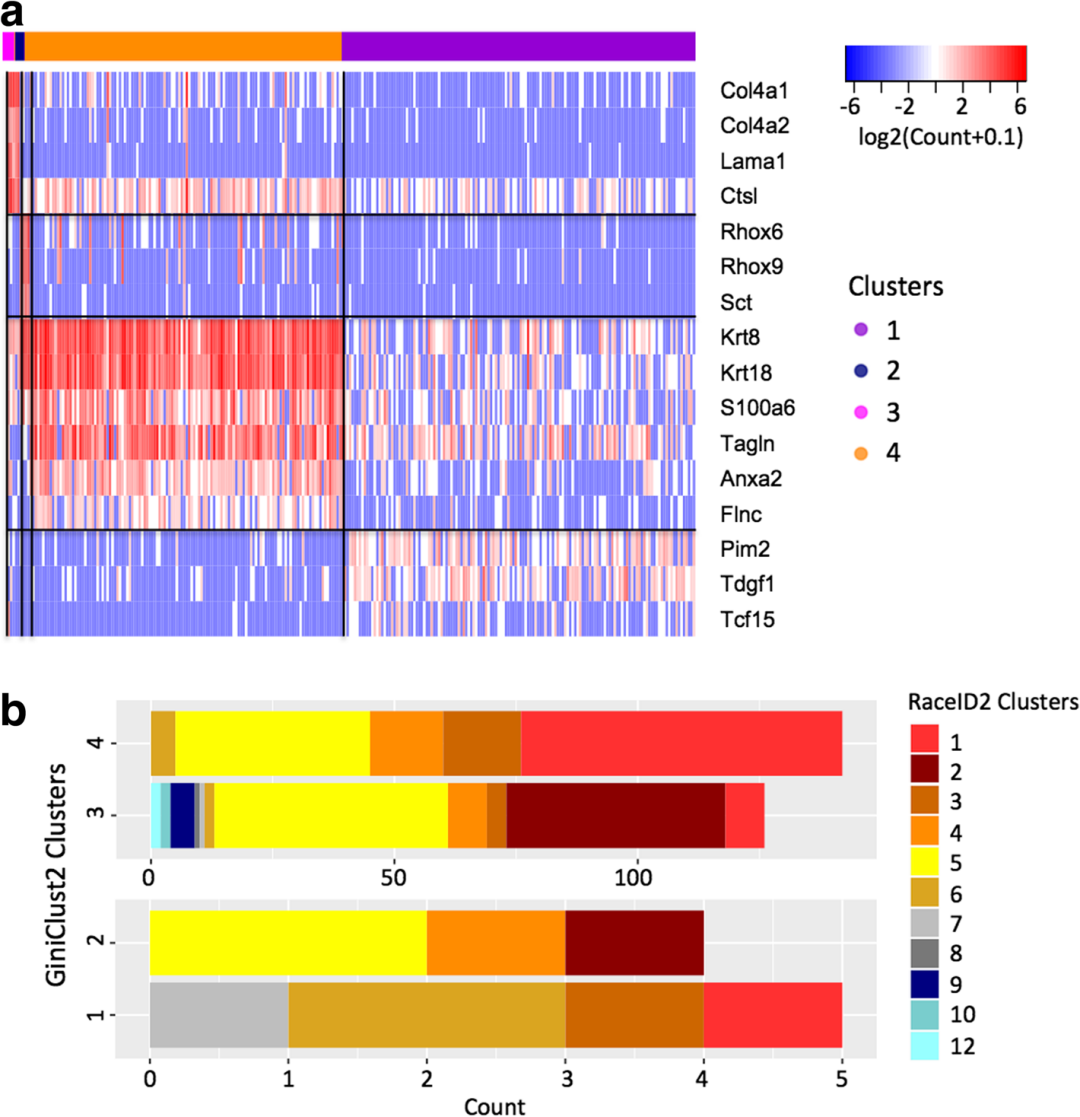
Analysis of the inDrop dataset for day 4 post-LIF mESC differentiation [19]. **a** A heatmap of top differentially expressed genes for each GiniClust2 cluster. The *color bar* above the heatmap indicates the cluster assignments. **b** A comparison of GiniClust2 and RaceID2 clustering results, for common (*above*) and rare (*below*) cell types. The same color-coding scheme is used in all panels

In addition, GiniClust2 identified two common clusters. The first group specifically expresses a number of genes related to cell growth and embryonic development, including *Pim2*, *Tdgf1*, and *Tcf15* (MAST likelihood ratio test *p* value < 1e-5, fold change > 2), indicating it corresponds to undifferentiated stem cells. The second group is strongly associated with a number of genes related to epiblast cells, including *Krt8*, *Krt18*, *S100a6*, *Tagln*, *Actg1*, *Anxa2*, and *Flnc* (MAST likelihood ratio test *p* value < 1e-5, fold change > 2), suggesting this group corresponds to an epiblast-like state. Of note, 114 of the 128 genes (Fisher exact test *p* value < 1e-88) specifically expressed in this group were selected as high Fano factor genes, confirming the utility of the Fano factor in detecting common cell types. Both populations were discovered in the original publication [19]. The dissimilarity between these cell types is evident in the heatmap (Fig. 4a) and composite tSNE plot (Additional file 2: Figure S5).

For comparison, we applied RaceID2 to analyze the same dataset. Unlike GiniClust2, RaceID2 broke each cluster into multiple subclusters, and failed to identify the rare cell clusters (Fig. 4b). With k = 2, RaceID2 found a total of 11 clusters. Clusters 1, 2, 4, and 9 display an epiblast-like signature, clusters 7 and 10 overexpress genes relating to maternal imprinting, and clusters 8 and 11 correspond to PrEn cells. From these results it appears that RaceID2 has difficulty in differentiating rare, biologically meaningful cell types from outliers.

### Scalability to large data sets

With the rapid development of single-cell technologies, it has become feasible to profile thousands or even millions of transcriptomes at single-cell resolution. Thus, it is desirable to develop scalable computational methods for single-cell data analysis. As a benchmark, we applied GiniClust2 to analyze the entire 68 k PBMC data set [17] described above to uncover hidden cell types. The complete analysis took 2.3 h on one core of a 2 GHz Intel Xeon CPU and utilized 237 GB of memory (not optimized for speed or memory usage). For comparison, RaceID2 analysis could not be completed for this large dataset. One possible explanation is this method may be limited to handling data sets with less than 65,536 data points due to an intrinsic vector size restriction in R. Our implementation of GiniClust2 circumvents this restriction by splitting up larger vectors into several smaller ones, with no changes to the functionality of the code. In principle, the same strategy can be implemented in RaceID2 to overcome this limitation. Comparisons of computational run-times between RaceID2 and GiniClust2 on smaller data sets show that the runtime of GiniClust2 scales better with the number of cells in the data set (Additional file 1, Additional file 2: Figure S6). For example, for a data set of 80 cells GiniClust2 and RaceID2 take the same amount of time, whereas for the simulated data set of 3023 cells GiniClust2 takes just under 10 min while RaceID2 takes 1 h and 13 mins. Despite the advantages of GiniClust2, it should be noted that GiniClust2 still requires a considerable amount of memory to run on very large data sets, presenting a limitation to the application of this method to even larger data sets.

**Fig. 5:**
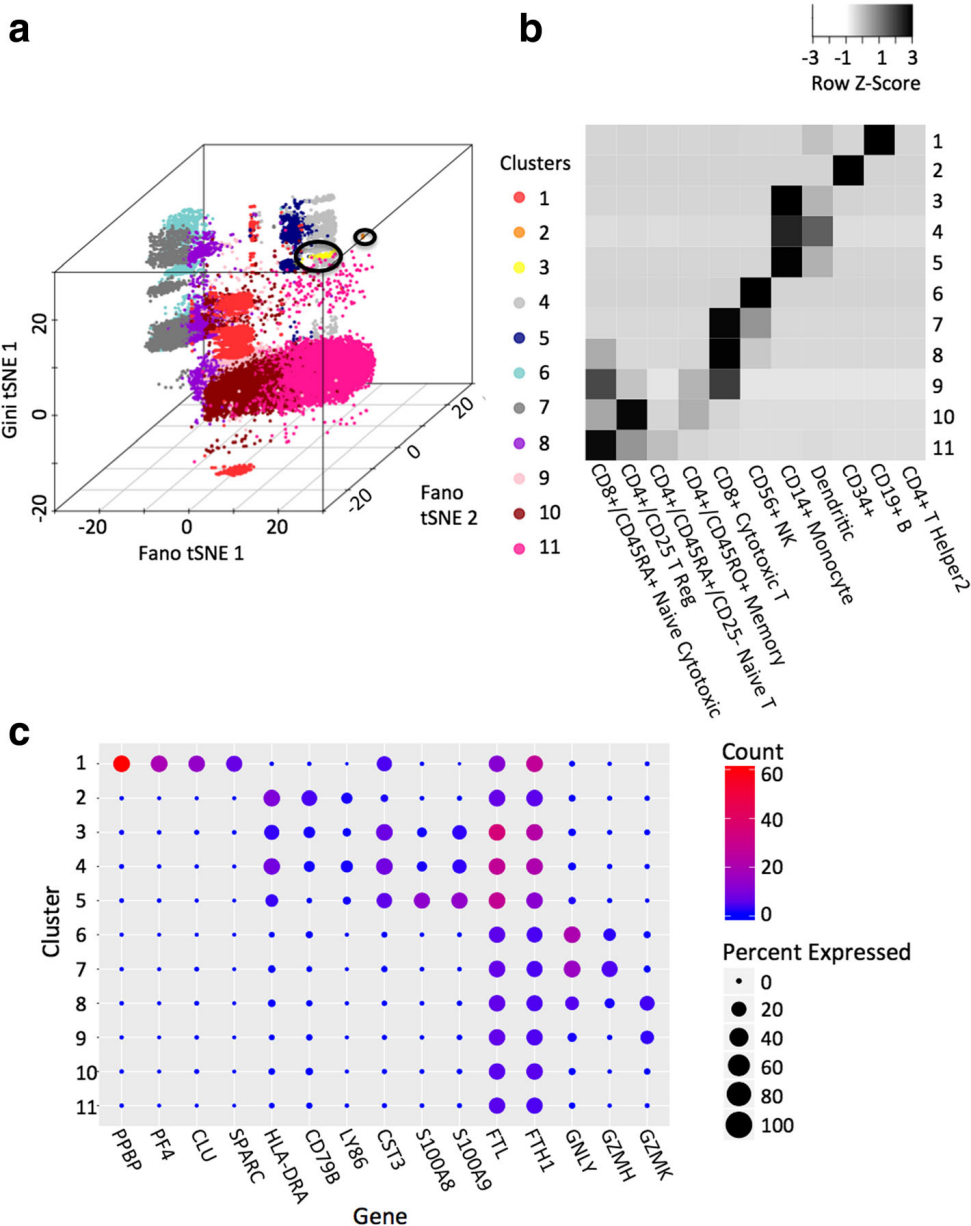
Results from the full 68 k PBMC data analysis. **a** A composite tSNE plot of the GiniClust2 results; rare cell types are circled. **b** A confusion map showing similarities between GiniClust2 clusters and reference labels. Values represent the proportion of cells per reference label that are in each cluster. **c** A bubble plot showing expression of cluster-specific genes. Size represents the percentage of cells within each cluster with non-zero expression of each gene, while color represents the average normalized UMI counts for each cluster and gene

Our analysis identified nine common clusters and two rare clusters (Fig. 5a). In general, the results of GiniClust2 and Fano factor-based k-means are similar; both agree well with the reference cell types (Fig. 5b**)**. To quantify this agreement, we use normalized mutual information (NMI), which is an entropy-based method normalized by cluster size that can be applied to multi-class classification problems [20]. A value of 1 indicates perfect agreement, whereas a value of 0 means the performance is as good as a random guess. Here, values are 0.540 for GiniClust2 and 0.553 for Fano factor-based k-means. Most of the discrepancy between the clustering results and reference labels are associated with T-cell subtypes. As noted by the original authors [17], these subtypes are difficult to separate because they share similar gene expression patterns and biological functions. The common clusters detected by GiniClust2 and Fano factor-based k-means express marker genes known to be specific to the cell types represented in the reference [21] (Fig. 5c).

With respect to rare cell types, our first group contains a homogeneous and visually distinct subset of 171 of 262 total CD34+ cells (cluster 2, Fig. 5a). This cluster was partially detectable using Fano factor-based k-means, although it was partially mixed with major clusters. The second rare cell cluster is previously unrecognized (cluster 3, Fig. 5a). This cluster contains 118 cells (0.17%) within a large set of 5433 immune cells with similar gene expression patterns. Among these 118 cells, 101 cells are classified as monocytes, whereas 16 are classified as dendritic cells, and one is classified as a CD34+ cell. Differential expression analysis (MAST likelihood ratio test *p* value < 1e-5, fold change > 2) identified 187 genes that are specifically expressed in this cell cluster, including a number of genes associated with tolerogenic properties, such as *Ftl*, *Fth1*, and *Cst3* [22], suggesting these cells may be associated with elevated immune response and metabolism. Additional validation would be necessary to determine whether this cluster is functionally distinct. Taken together, these results strongly indicate the utility of GiniClust2 in analyzing large single-cell datasets.

## Discussion and conclusions

According to the “no free lunch” theorem [23], an algorithm that performs well on a certain class of optimization problems is typically associated with degraded performance for other problems. Therefore, it is expected that clustering algorithms optimized for detecting common cell clusters are unable to detect rare cell clusters, and vice versa. While ensemble clustering is a promising strategy to combine the strengths of multiple methods [4, 5, 16], our analysis shows that the traditional, unweighted approach does not perform well.

To optimally combine the strengths of different clustering methods, we have developed GiniClust2, which is a cluster-aware, weighted ensemble clustering method. GiniClust2 effectively combines the strengths of Gini index- and Fano factor-based clustering methods for detecting rare and common cell clusters, respectively, by assigning higher weights to the more reliable clusters for each method. By analyzing a number of simulated and real scRNA-seq datasets, we find that GiniClust2 consistently performs better than other methods in maintaining the overall balance of detecting both rare and common cell types. This weighted approach is generally applicable to a wide range of problems.

GiniClust2 is currently the only rare cell-specific detection method equipped to handle such large data sets, as demonstrated by our analysis of the 68 k PBMC dataset from 10X Genomics. This property is important for detecting hidden cell types in large datasets, and may be particularly useful for annotating the Human Cell Atlas [24].

## Methods

### Data preprocessing

The processed mouse ESC scRNA-seq data are represented as UMI filtered-mapped counts. Removing genes expressed in fewer than three cells, and cells expressing fewer than 2000 genes, we were left with a total of 8055 genes and 278 cells.

The processed 68 k PBMC dataset, represented as UMI counts, was filtered and normalized using the code provided by 10X Genomics (https://github.com/10XGenomics/single-cell-3prime-paper). The resulting data consist of a total of 20,387 genes and 68,579 cells. Cell-type labels were assigned based on the maximum correlation between the gene expression profile of each single cell to 11 purified cell populations, using the code provided by 10X Genomics.

### GiniClust2 method details

The GiniClust2 pipeline contains the following steps.

#### Step 1: Clustering cells using Gini index-based features

The Gini index for each gene is calculated and normalized as described before [11]. Briefly, the raw Gini index is calculated as twice the area between the diagonal and the Lorenz curve, taking a range of values between 0 and 1. Raw Gini index values are normalized by removing the trend with maximum expression levels using a two-step LOESS regression procedure as described in [11]. Genes whose normalized Gini index is significantly above zero (*p* value < 0.0001 under the normal distribution assumption) are labeled high Gini genes and selected for further analysis.

A high Gini gene-based distance is calculated between each pair of cells using the Jaccard distance metric. This is used as input into DBSCAN [25], which is implemented using the dbscan function in the fpc R package, with method = “dist”. Parameter choices for eps and MinPts are discussed in Additional file 1.

#### Step 2: Clustering cells using Fano factor-based features

The Fano factor is defined as the variance over mean expression value for each gene. The top 1000 genes are chosen for further analysis. Principal component analysis (PCA) is applied to the gene expression matrix for dimensionality reduction, using the svd function in R. The first 50 principal components are reserved for clustering analysis. Cell clusters are identified by k-means clustering, using the kmeans function in R with default parameters. Optimal choice of k is discussed in Additional file 1. To improve robustness, 20 independent runs of k-means clustering with different random initializations are applied to each dataset, and the optimal clustering result is selected.

#### Step 3. Combining the results from steps 1 and 2 via a cluster-aware, weighted ensemble approach

We adapted the weighted consensus clustering algorithm developed by Li and Ding [13] by further considering cluster-specific weighting. For GiniClust, higher weights are assigned to the rare cell clusters and lower weights to common clusters, whereas the opposite scheme is used to weight the outcome from Fano factor-based k-means clustering. This allows us to combine the strengths of each clustering method. The mathematical details are described as follows, and visualized in Fig. 1b.

Let *P*^*G*^ be the partitioning provided by GiniClust, and *P*^*F*^ the partitioning provided by Fano factor-based clustering. Each partition consists of a set of clusters: ${C}^G={C}_1^G,{C}_2^G,\dots, {C}_{k_G}^G$, and ${C}^F={C}_1^F,{C}_2^F,\dots, {C}_{k_F}^F.$ Define the connectivity matrices as:

$$ {M}_{ij}\left({P}^G\right)=\left\{{\displaystyle \begin{array}{c}1,\left(i,j\right)\in {C}_k\left({P}^G\right)\\ {}0, otherwise\end{array}}\operatorname{},\mathrm{and}\kern0.28em {M}_{ij}\left({P}^F\right)=\right\{{\displaystyle \begin{array}{c}1,\left(i,j\right)\in {C}_k\left({P}^F\right)\\ {}0, otherwise.\end{array}}\operatorname{} $$

If two cells are clustered together in the same group, their connectivity is 1, while if they are clustered separately, their connectivity is 0. Define the weighted consensus association as:

$$ \overline{M_{ij}}={w}_{ij}^G{M}_{ij}\left({P}^G\right)+{w}_{ij}^F{M}_{ij}\left({P}^F\right) $$

where ${w}_{ij}^G+{w}_{ij}^F=1,{w}_{ij}^G,{w}_{ij}^F\ge 0\forall i,j\in \left[1,n\right]$, *n* represents the number of cells. Weights ${w}_{ij}^G$ and ${w}_{ij}^F$ are specific to each pair of cells, and are determined based on ${\overset{\sim }{w}}_i^G$ and ${\overset{\sim }{w}}_i^F$, weights that are specific to each cell.

For simplicity, we set the cell-specific weights for the Fano factor-based clusters as a constant: ${\overset{\sim }{w}}_i^F={f}^{\prime }$. The cell-specific GiniClust (${\overset{\sim }{w}}_i^G\Big)$ weights are determined as a function of the size of the cluster containing the particular cell. Our choices for these weights derive from the observation that as the proportion of the rare cell type increases, the utility of GiniClust begins to decline. For simplicity, we model the cell-specific GiniClust weights using a logistic curve, specified by the following function:

$$ {\overset{\sim }{w}}_i^G\left({x}_i\right)=1-\frac{1}{1+{e}^{-\frac{x_i-{\mu}^{\prime }}{s^{\prime }}}} $$

where *x*_*i*_ is the proportion of the GiniClust cluster to which cell i belongs, *μ'* is the rare cell type proportion at which GiniClust and Fano factor-based clustering methods have approximately the same ability to detect rare cell types, and *s'* represents how quickly GiniClust loses its ability to detect rare cell types above *μ'*. The parameters *s'*, *μ'*, and *f'* can be viewed as intermediate variables that are closely associated with the parameters *s*, *μ*, and *f*, schematically shown in Fig. 1c**.** Specifically, $f=\frac{f^{\prime }}{1+{f}^{\prime }}$, *s* = *s'*, and *μ* is obtained relative to the other parameters through the following relationship: ${f}^{\prime }=1-\frac{1}{1+{e}^{-\frac{\mu -{\mu}^{\prime }}{s^{\prime }}}}$. The selection of the parameter values for *s'*, *μ'*, and *f'*, as well as a sensitivity analysis, are described in Additional file 1.

To set the cell pair-specific weights, we first define

$$ {\tilde{w}}_{ij}^G=\max \left({\tilde{w}}_i^G,{\tilde{w}}_j^G\ \right)\;\mathrm{and}\;{\tilde{w}}_{ij}^F={\tilde{w}}_i^F $$

Then, weights are normalized to 1:

$$ {w}_{ij}^G=\frac{{\tilde{w}}_{ij}^G}{{\tilde{w}}_{ij}^G+{\tilde{w}}_{ij}^F}\;\mathrm{and}\;{w}_{ij}^F=\frac{{\tilde{w}}_{ij}^F}{{\tilde{w}}_{ij}^G+{\tilde{w}}_{ij}^F} $$

Each cell–cell pair will thus be assigned a weighted consensus association between 0 and 1, which is a weighted average of both GiniClust and Fano factor-based clustering associations, where the weights are functions of the size of the cell clusters.

At this point, the weighted consensus association matrix provides a probabilistic clustering for each cell, where each entry represents the probability that cell i and cell j reside in the same cluster. To transform this into a final deterministic clustering assignment, we optimize the following:

$$ {\mathit{\min}}_U{\left|\left|\overline{M}-U\right|\right|}^2, $$

where *U* is any possible connectivity matrix. In Li and Ding [13], this optimization problem is solved via symmetric non-negative matrix factorization (NMF) to yield a soft clustering. To obtain a hard clustering we add an orthogonality constraint, leading to k-means clustering [26], implemented once again using the kmeans R function.

### tSNE visualization

Dimension reduction by tSNE [27] is performed using the Rtsne R package. The tSNE algorithm is first run using the Gini-based distance to obtain a one-dimensional projection of each cell. For large data sets, tSNE is run on the first 50 principal components of the Gini-based distance to prevent tSNE from becoming prohibitively slow. Then, the tSNE algorithm is run using the first 50 principal components of our Fano-based Euclidean distance to obtain a separate two-dimensional projection. The three resulting dimensions (one for Gini-based distance and two for Fano-based distance) are plotted to visualize cluster separation.

### Differential expression analysis on resulting clusters

Differentially expressed genes for each cluster are determined by comparing their gene expression levels to all other clusters. This is performed using the zlm.SingleCellAssay function in the R MAST package [28], with method = “glm”. *P* values for differentially expressed genes are calculated using the lrTest function, with a hurdle model.

### SC3 analysis

SC3 [4] was accessed through the SC3 Bioconductor R package. SC3 was applied to the simulated data set post-filtering using default parameters, with k = 6 to match the true number of clusters. The author-recommended choice of k using the Tracy-Widom test yielded a k of 55, and was deemed inappropriate for this analysis.

### CSPA analysis

Matlab code for the CSPA [16] was accessed through http://strehl.com/soft.html, under “ClusterPack_V2.0.” CSPA was applied to the Gini and Fano-based clustering results for the simulated data set, using the clusterensemble function, specifying the CSPA option. Results are shown for k = 5, the default parameter, and k = 6, the true number of clusters.

### RaceID2 analysis

RaceID2 [10] R scripts were accessed through https://github.com/dgrun/StemID. RaceID2 was applied to already-filtered data sets as above to make results directly comparable to GiniClust2, with default parameters. Results are shown for k set to the default parameter as determined by a within-cluster dispersion saturation metric [10], and k set to match the corresponding GiniClust2 k parameter specification.

### Hierarchical clustering analysis

Hierarchical clustering was performed on a Fano-based Euclidean distance using the hclust function in R. For the simulated data analysis, results are shown for choices k = 6, to match the true number of clusters, and k = 2, the parameter value as determined by the gap statistic through the clusGap function in R. For the subsampled PBMC analysis, results are shown for k = 3, to match the true number of clusters.

### Community detection analysis

Community detection was performed on a k-nearest neighbor (kNN) graph, using a high Fano feature space, for simulated and subsampled data sets. Function nn2 in the RANN R package was used to compute a kNN distance with default parameters. The igraph R package was used to perform community detection, using the cluster_edge_betweenness function with default parameters.

### Simulation details

We created synthetic data following the same approach as Jiang et al. [11], specifying one large 2000 cell cluster, one large 1000 cell cluster, and four rare clusters of 10, 6, 4, and 3 cells, respectively. Gene expression levels are modeled using a negative binomial distribution, and distribution parameters are estimated using an intestinal scRNA-seq data set using a background noise model as in Grün et al. [9]. To create clusters with distinct gene expression patterns, we permute 100 lowly (mean < 10 counts) and 100 highly (mean > 10 counts) expressed gene labels for each cluster (see Jiang et al. [11] for more details). This results in a 23,538 gene by 3023 cell data set. After filtering (as above) we are left with 3708 genes and 3023 cells.

### 10X Genomics data subsampling

The full 68 k 10X Genomics PBMC dataset is down-sampled for model evaluation. We consider only three cell types here. CD19+ B cells are defined by their correlation to reference transcriptomes as in Zheng et al. [17]. CD14+ monocytes and CD56+ NK cells are defined in the same way, but here we recognize that these broadly defined cell types actually consist of two subtypes each. We therefore use additional known markers to refine each cell type definition. With regard to CD14+ monocytes, we use macrophage markers *Cd68* and *Cd37* [21] to separate macrophages and monocytes, and we define macrophage cells as those with positive expression of both markers. These cells are selected for subsampling. The CD56+ NK cells are composed of NK and NKT cells, so we use T-cell markers *Cd3d*, *Cd3e*, and *Cd3g* [21] to separate the groups, and define the NK cells as those with zero expression of these three markers. There is some additional heterogeneity in this NK group, so we choose to include only those NK cells that were most highly correlated (top 50%) to the reference transcriptomes. Given these cell type definitions, we created seven sets of 20 subsampled data sets each for a total of 140 data sets in the following manner: five cells were randomly sampled from the macrophage cell population to form a “rare” cell group for all 120 datasets. Then, for each set of 20 data sets, cells were randomly sampled from the NK and B cells in specified numbers to form “common” cell clusters, the details of which are listed in Additional file 2: Table S1.

## Additional files

Additional file 1: Supplementary information. (DOCX 38 kb)

Additional file 2: Supplementary Figures S1–S10, Supplementary Table S1. (PDF 1509 kb)

## Abbreviations

ARI: Adjusted rand index
CSPA: Cluster-based similarity partitioning algorithm
DBSCAN: Density-based spatial clustering of applications with noise
kNN: k-Nearest neighbor
LIF: Leukemia inhibitory factor
MAST: Model-based analysis of single-cell transcriptomics
MCC: Matthews correlation coefficient
mESC: Mouse embryonic stem cell
NK: Natural killer
NMF: Non-negative matrix factorization
NMI: Normalized mutual information
PBMC: Peripheral blood mononuclear cell
PCA: Principal component analysis
PrEn: Primitive endoderm
RaceID: Rare cell type identification
scRNA-seq: Single-cell RNA-sequencing
tSNE: t-Distributed stochastic neighbor embedding
