# Supplementary material for: GiniClust2: a cluster-aware, weighted ensemble clustering method for cell-type detection
Source: Genome Biol. 2018 May 10;19:58. doi: 10.1186/s13059-018-1431-3 (PMC5946416; doi:10.1186/s13059-018-1431-3)
Supplement: Supplementary file 1 — Supplementary information. (DOCX 38 kb) [file 13059_2018_1431_MOESM1_ESM.docx]

**SUPPLEMENTAL INFORMATION**

**Evaluation of clustering performance using additional metrics**

Several additional metrics are used to compare clustering accuracy across various clustering methods, for both simulated and subsampled data. Results for simulated data are shown in **Additional file 2: Fig. S1**, and results for a subset of the subsampled data sets (corresponding to a rare cell type of 1.6%) are shown in **Additional file 2: Fig. S4**. Full results for the subsampled data are not shown for reasons of brevity, but results for this particular rare cell type proportion are representative of the overall results. The additional metrics used to evaluate clustering accuracy are: purity, normalized mutual information (NMI), micro-averaged F-measure, adjusted rand index (ARI), and entropy. These various metrics are introduced to give a more complete view of the clustering results, as each method measures accuracy in a slightly different way. Purity is a measure of how often a cluster contains a single cell type, where 1 indicates that all clusters contain a single cell type, and a value of 0 indicates poor clustering [33]. Unfortunately, this metric does not penalize for overclustering, and a perfect clustering can be achieved by clustering each cell separately. Often, more complex metrics are required. NMI is an entropy-based method normalized by cluster size [20], where a value of 1 indicates perfect agreement, whereas a value of 0 means the performance is as good as random guess. The micro-averaged F-measure is the harmonic mean of micro-averaged precision and recall rates, which are computed by summing true positive, true negative, false positive and false negative values over all cell types. Values also range from 0 to 1, with values close to 1 implying better clustering [34]. The ARI is a version of the rand index that is corrected for chance, and also takes into account both false positives and negatives. A value of 1 indicates a perfect clustering, a value of 0 is the expected value for a random clustering, and the metric takes on negative values if the clustering is worse than expected [35]. Entropy is a measure of disorder within the clustering results that ranges from 0 to 1, where values close to 0 imply less disorder and better clustering [36]. As this is the only metric where a lower value means a better clustering, we show 1-entropy for a more intuitive visualization.

**Results for a naïve combination of high Fano and Gini genes in simulated data**

The most obvious approach to combining the superior performances of Gini and Fano-based feature spaces for detecting rare and common cell types, respectively, may be to combine the two feature spaces, and perform clustering on this combined space. **Additional file 2: Fig. S3** shows a two-dimensional tSNE of the Jaccard distance of this combined feature space, colored with the true cell clusters, followed by clustering results on this same space using DBSCAN and k-means clustering methods. Neither of these clustering methods is able to recapitulate all six clusters, and the visualization gives an indication as to why. The two larger clusters are visually separable, but the smaller clusters are in close proximity to the largest cluster, and are indistinguishable from each other. This combination of two distinct feature spaces is undesirable because it dilutes the signal from each feature space, and further demonstrates the need for a consensus clustering approach.

**Parameter choice details**

*Parameter choice for DBSCAN*

DBSCAN has two parameters: MinPts and eps. MinPts is specified as 3 for all data sets except for the PBMC data, where MinPts is set to 100, in accordance with the larger size of this data set. This corresponds to the minimum cluster size for which we would expect to see a biologically relevant cluster. In general, we find an appropriate MinPts specification to be about 0.1% of the total number of cells.

The eps parameter is determined by a k-nearest-neighbors (kNN) plot as recommended by the authors of DBSCAN [25]. According to their approach, distance from each point to its kth nearest neighbor is plotted in ascending order, where k=MinPts. This will form a line featuring an inflection point, at which lies the recommended choice for eps [25]. If multiple inflection points exist, this may suggest that multiple values of eps are worth exploring; however, in our case as we are concerned with rare clusters, we only consider the smallest choice of eps, corresponding to the first inflection point. Here, our Gini-based distance metric is particularly low-dimensional due to the use of Jaccard distance and a small number of high Gini genes. This causes cells with similar expression profiles to have pairwise distances of zero, which distorts the traditional kNN-distance curve shape and makes the inflection point harder to visualize (**Additional file 2: Fig. S7**). We provide an alternate numerical approach to approximating the inflection point: after removing all zero distances, the inflection point roughly corresponds to the kNN-distance of the (0.00125*total number of cells*MinPts)^th^ cell. For the 68k data set, the computation of all kNN distances was prohibitive, so we subsampled 2057 cells and computed 3-NN distances to maintain the ratio of MinPts to the total sample size.

*Choice of k for k-means clustering*

We give the option of automatically determining k using the gap statistic. However, observing differentially expressed genes and visualizing k-means clusters gives the best intuition as to the optimal k. We also do not suggest using the gap statistic for large data sets due to its computational demands. For the simulated data, we chose k=2, in accordance with both the number of large clusters and the gap statistic, but show that k=3 will also yield the same result (**Additional file 2: Fig. S2**). For subsampled PBMC data sets, k was chosen as 2 or 3 depending on the ability of k-means to pick up the rare NK cell group. k was chosen as 2 for the day 4 post-LIF mouse embryonic stem cell data as we found this number to best group biologically meaningful cell types.

For the 68k PBMC data, we chose k=10 to allow for direct comparison with clustering results from Zheng et al. [17]. For comparison to the k=10 parameter choice, we additionally show results for both choices k=8 and k=12 for the Fano-based clustering step (**Additional file 2: Fig. S8**). All parameter choices perform comparably, with NMI values of 0.542, 0.541 and 0.498, respectively, when compared to the reference labels. The k=8 clustering results in two fewer clusters within the CD56+ NK, CD8+ Cytotoxic T, CD8+/CD45RA+ Naïve Cytotoxic continuum. The k=12 clustering further splits clusters containing overlapping CD8+/CD45RA+ Naïve Cytotoxic, CD4+/CD45RA+/CD25- Naïve T, CD4+/CD25 T Reg, and CD4+/CD45RO+ Memory cells, as well as adding another cluster to the aforementioned CD56+ NK, CD8+ Cytotoxic T, CD8+/CD45RA+ Naïve Cytotoxic continuum. These changes are minor as they occur predominantly in regions of unclear identity.

*Parameter choices for weighted consensus clustering*

As discussed in the Materials and Methods section, the parameter values for$\mu$, $s$, and $f$ are derived through intermediate variables $\mu'$, $s'$, and $f'$. The values for these intermediate variables are determined empirically using the following procedure. First, we set $\mu'$= 4*(MinPts/total number of cells), where MinPts represents the minimum cluster size allowed by DBSCAN. We find that this is the approximate cell fraction where GiniClust and Fano-factor-based clustering perform equally. Next, using the same logic, we set the value for$s'$ such that the 99^th^ percentile of the GiniClust weighting distribution is reached at 6*(MinPts/total number of cells). We find that this is the approximate cell fraction in which GiniClust can no longer detect the rare cell type. Finally, we set $f'$ = 0.1. While these parameter settings cannot guarantee optimal performance, results from our sensitivity analysis (see next section) strongly suggest that the clustering results are robust over a wide range of parameter values.

**Sensitivity analysis on simulated data**

GiniClust2 parameters were varied one at a time on simulated data to test the robustness of the method to specific parameter choices. The following parameters were varied: DBSCAN parameters MinPts and eps, k-means parameter k, Gini and Fano gene thresholds, and weighting scheme parameters $\mu$, $s$, and $f$. In addition, the behavior of GiniClust2 across various signal:noise ratios was evaluated by running the method on several variations of the original data set. The noise level was simulated by varying the scale parameter of the generative negative binomial distribution (see **Methods**). Clustering accuracy was evaluated using several metrics: NMI, ARI, entropy, purity, and the micro-averaged F-measure. Results of these analyses are shown in **Additional file 2: Fig. S9**.

Our analysis suggests that the clustering results are strongly affected by the choice of k. A small k results in combining the large clusters, while a large k results in splitting the large clusters into smaller subgroups. This resolution uncertainty is intrinsic to all clustering methods. For all other parameter changes, metrics do not dip below 0.96, indicating the robustness of the clustering results to these parameter choices. Perhaps more importantly, clustering results are perfect over a wide range of many of these parameter values.

**Analysis of the 10X Genomics data supports a logistic function model**

We test whether the consensus clustering weighting function $w_{i}^{G}(x_{i})$ accurately represents the power of GiniClust to detect rare cell types over a range of cell type proportions. In the **Results** section we discuss a subsampling analysis performed by selecting macrophage, NK and B cells at varying proportions from a 10X Genomics dataset consisting of about 68,000 peripheral blood mononuclear cells (PBMCs) [17]. Cells are classified based on transcriptomic similarity with purified cell-types and additional known gene markers (see **Methods** for full details). Cell types are sampled 140 times according to **Additional file 2: Table S1** such that the rare macrophage group ranges in cell type proportion from 0.2% to 11.6%.

To capture the power of GiniClust and Fano-factor-based k-means to detect cell types of varying rarity, we define “detection” of the rare cell type as the clustering together of at least 3 out of the 5 rare cells, while including at most 2 other cells in this rare group. For the subsampled PBMC data we calculate the rare cell type detection rates of both GiniClust and Fano-factor-based k-means for each of the rare cell type proportions (**Additional file 2: Fig. S10a**). We next calculate the ratio between the GiniClust detection ability and the sum of both GiniClust and Fano-factor-based k-means detection abilities (**Additional file 2: Fig. S10b**). This is a measure of the ability of GiniClust over Fano-factor-based k-means in detecting the rare cell type, which we tried to capture in our GiniClust weighting function. We can see that the shape of the curve in **Additional file 2: Fig. S10b** closely mimics that of the logistic GiniClust weighting function, also pictured in **Additional file 2: Fig. S10b**, and suggests that such a logistic function shape is appropriate for defining GiniClust weights.

**Comparison of the computational performance of GiniClust2 and RaceID2**

GiniClust2 and RaceID2 were run on all smaller data sets (<=3023 cells) using a 2.5 GHz Intel Core i7 CPU with 16 GB memory. Runtimes for these methods are shown in **Additional file 2: Fig. S6**. Both methods were run using default—and where applicable, automatic—parameter choices. For datasets above 155 cells, GiniClust2 is faster than RaceID2, and scales better than RaceID2 for an increasing number of cells. Only GiniClust2 can be run for very large data sets (68k cells), and therefore, a comparison cannot be shown. It should be noted that for these large data sets, a few code alterations (see **Methods**) make running GiniClust2 a faster process without sacrificing accuracy, and the runtime does not scale with the runtimes for these smaller data sets.

Additional References:

33. Manning CD, Raghavan P, Schütze H. Introduction to information retrieval: Cambridge University Press; 2008.

34. Van Rijsbergen CJ. Information retrieval: Butterworths; 1979.

35. Hubert L, Arabie P. Comparing partitions. J Classif. 1985;2:193–218.

36. Tolman RC. Principles of statistical mechanics: Courier Corporation; 1938.
