# Supplementary material for: GiniClust2: a cluster-aware, weighted ensemble clustering method for cell-type detection
Source: Genome Biol. 2018 May 10;19:58. doi: 10.1186/s13059-018-1431-3 (PMC5946416; doi:10.1186/s13059-018-1431-3)
Supplement: Supplementary file 2 — Supplementary Figures S1–S10, Supplementary Table S1. (PDF 1509 kb) [file 13059_2018_1431_MOESM2_ESM.pdf]

Supplementary Material

Daphne Tsoucas[1,2,*], Guo-Cheng Yuan[1,2]

1. Department of Biostatistics and Computational Biology, Dana-Farber Cancer Institute, Boston, MA 02115, USA

2. Department of Biostatistics, Harvard T.H. Chan School of Public Health, Boston, MA 02115, USA

*corresponding author

Emails: dtsoucas@g.harvard.edu; gcyuan@jimmy.harvard.edu

1. Supplementary Figures

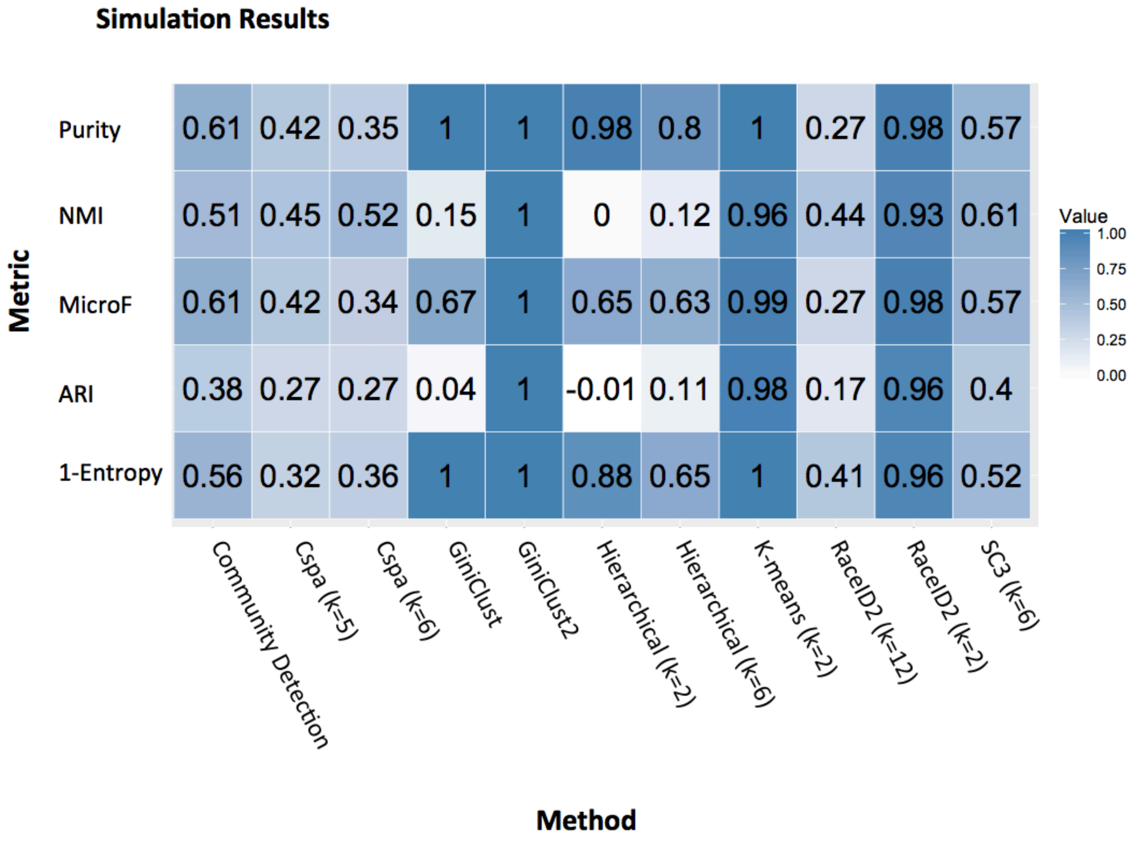

Figure S1. A summary of the clustering results of GiniClust2, RaceID2, and other comparable methods on simulated data. Clustering accuracy is measured using several metrics: purity, normalized mutual information (NMI), micro-averaged F-measure, adjusted rand index (ARI), and entropy.

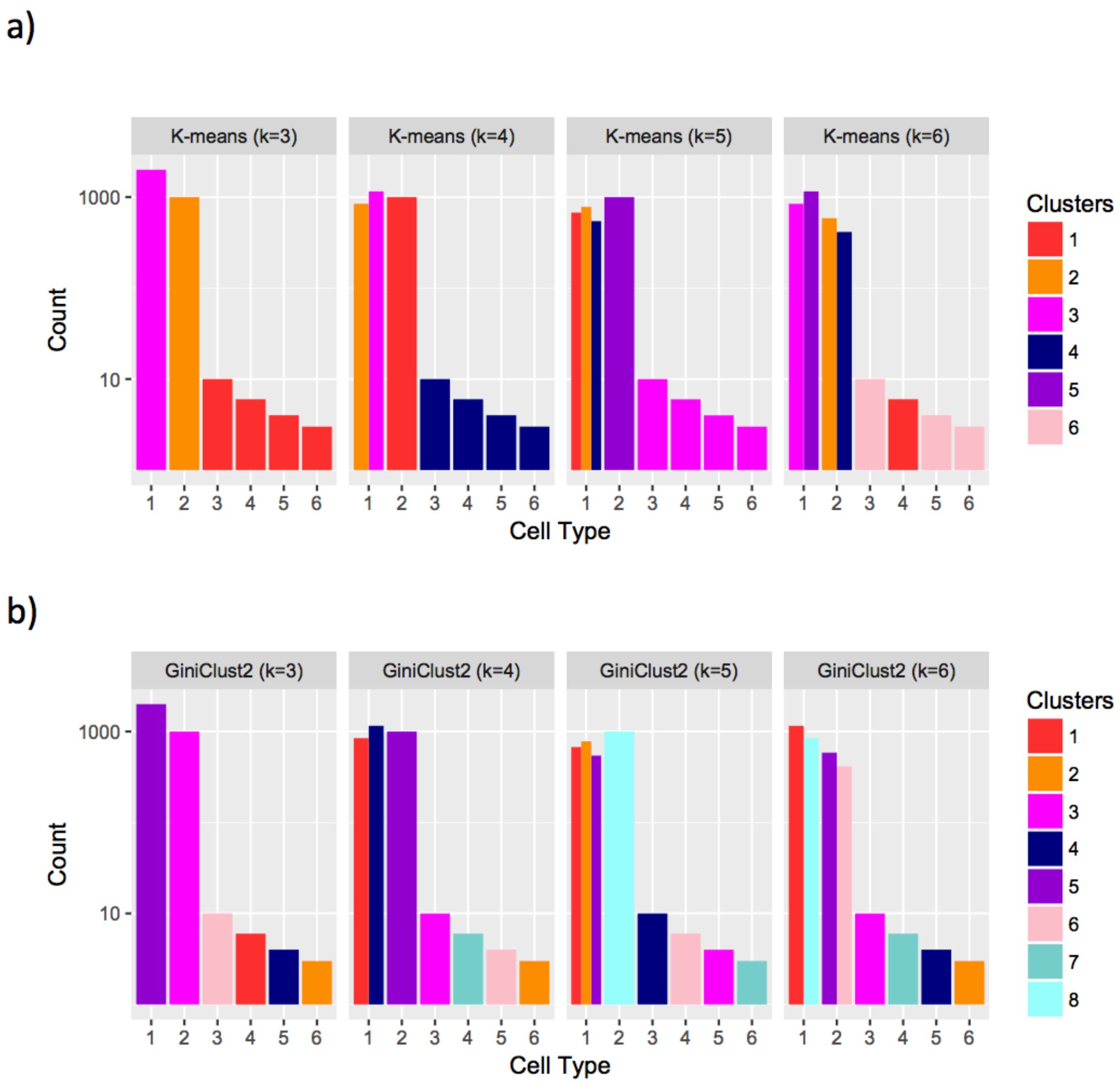

Figure S2. The effect of various choices of k on (a) the k-means step and (b) the overall clustering of our GiniClust2 method for the simulated data. Each bar represents the contribution of a cluster to the total number of cells in each reference type.

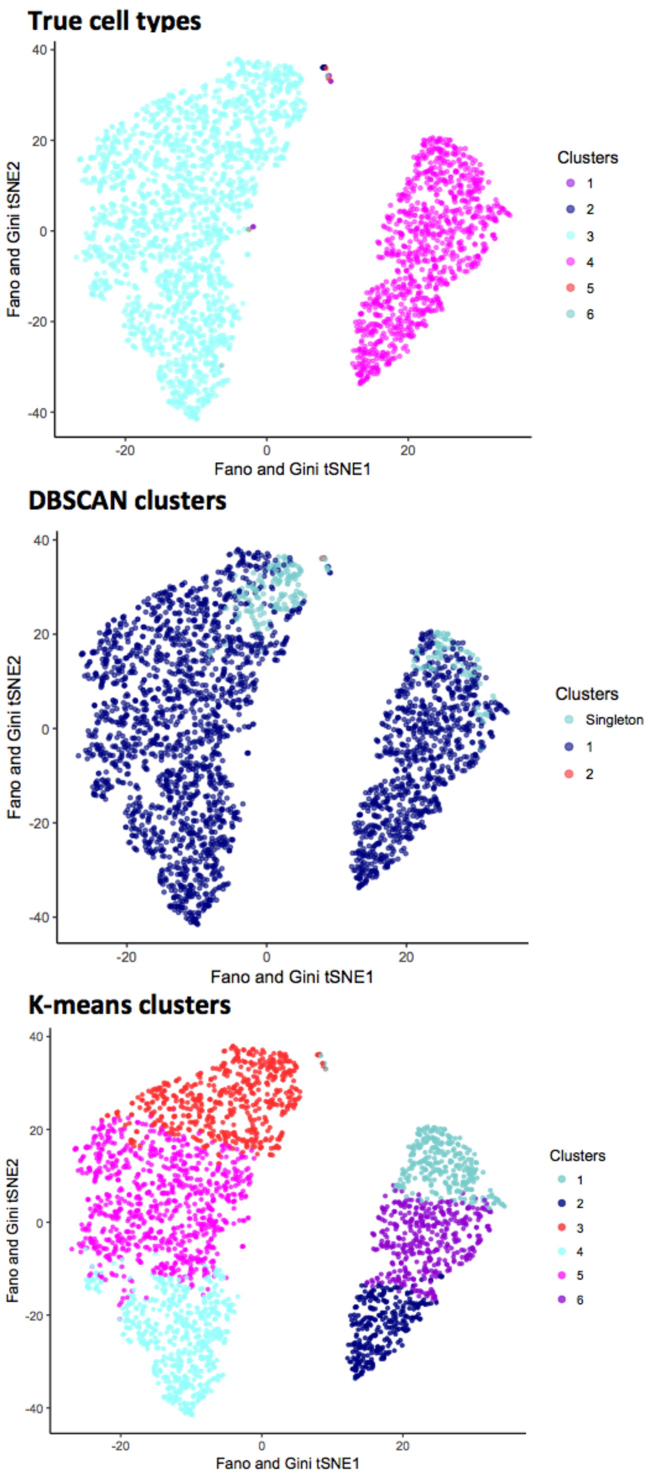

Figure S3. Two-dimensional tSNE representations of the simulated data, using a feature space based on a naïve combination of high Gini and Fano genes. Colors represent the true cell types, followed by clustering results using DBSCAN and k-means clustering methods on this naïve feature space, respectively.

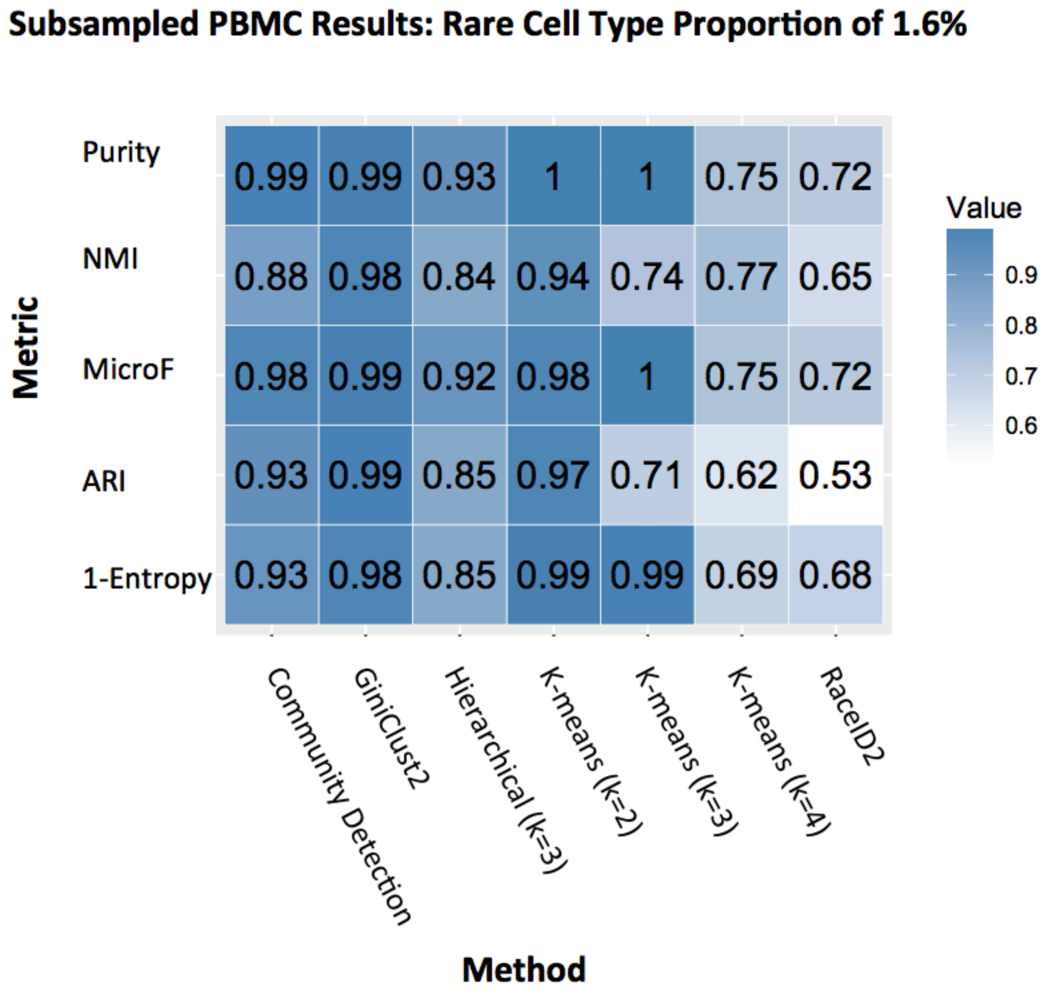

Figure S4. Clustering results for a subset of the subsampled PBMC data sets containing a representative rare cell type proportion of 1.6%, for GiniClust2, RaceID2, and other comparable methods. These were measured using purity, normalized mutual information (NMI), micro-averaged F-measure, adjusted rand index (ARI), and entropy.

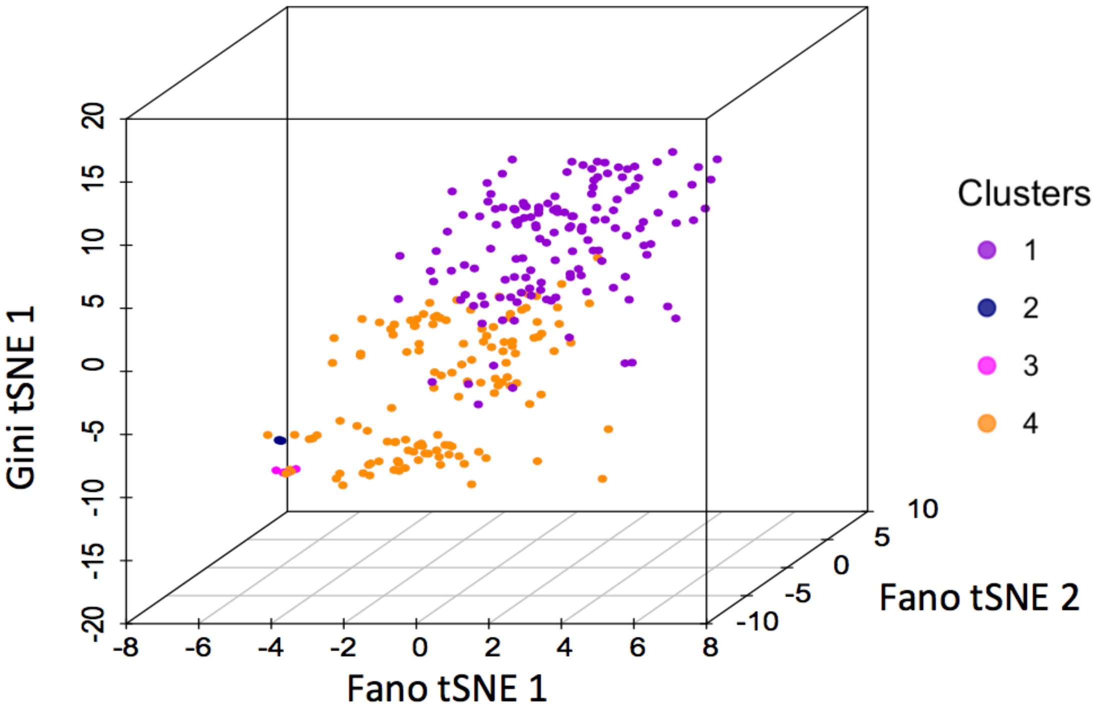

Figure S5. A composite tSNE plot representing the GiniClust2 clustering results for the inDrop dataset for day 4 post-LIF mESC differentiation [19].

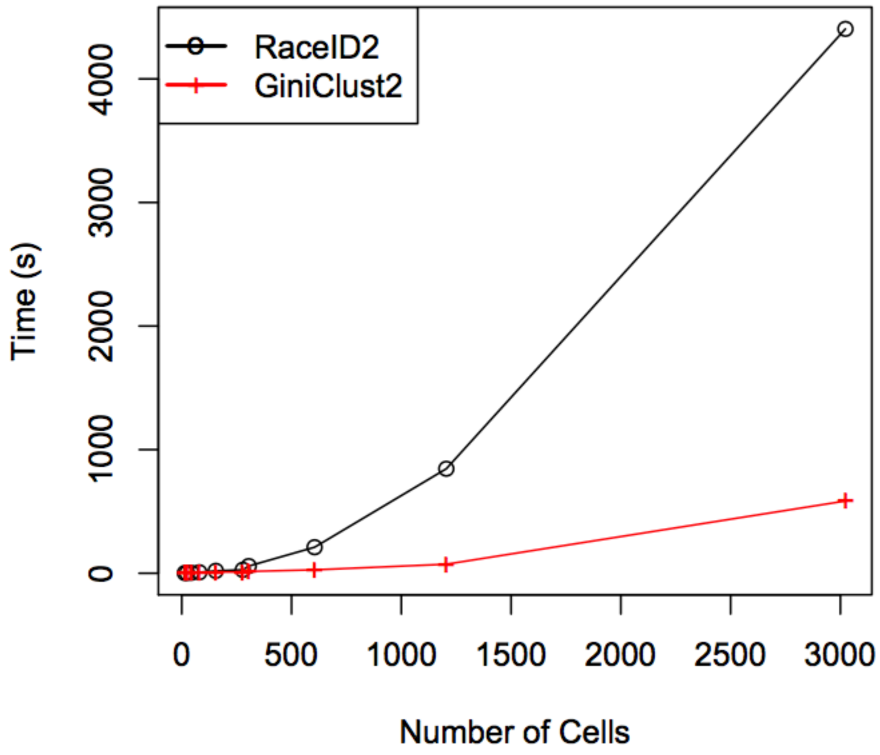

Figure S6. A comparison of computational runtimes for GiniClust2 and RaceID2, for data sets ranging from 43 to 3023 cells. The methods were run on a 2.5 GHz Intel Core i7 CPU with 16 GB memory.

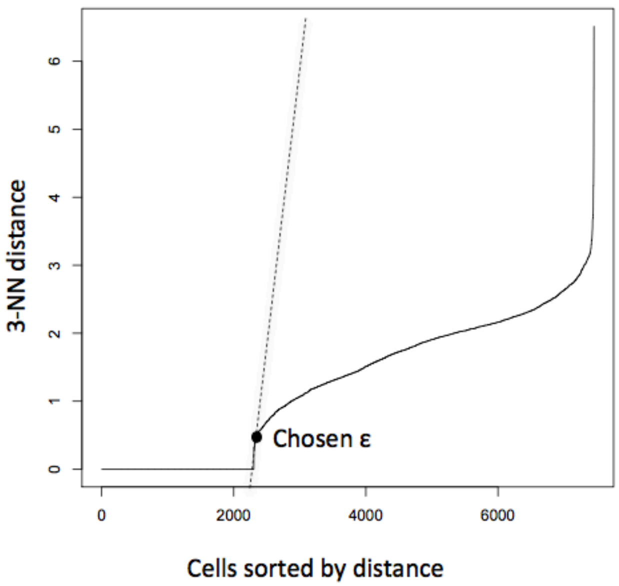

Figure S7. An illustration of the eps parameter selection process for DBSCAN, the clustering method used in GiniClust. Eps is chosen as the distance at the inflection point in the k-nearest-neighbors distance plot.

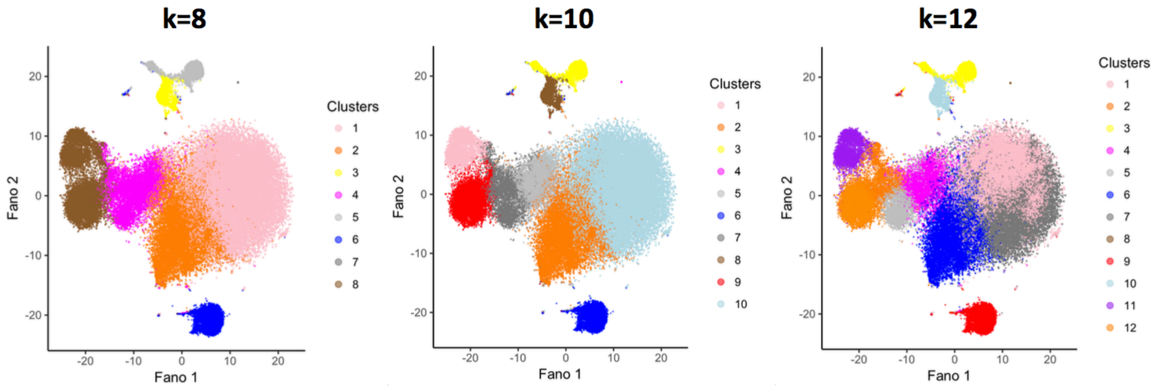

Figure S8. The effect of various choices of k on the Fano-factor-based k-means step of GiniClust2 for the full 68k PBMC data. Clustering results for Fano-factor-based kmeans using k=8, k=10, and k=12, respectively, are shown using three two-dimensional tSNE plots colored with each set of corresponding cluster labels.

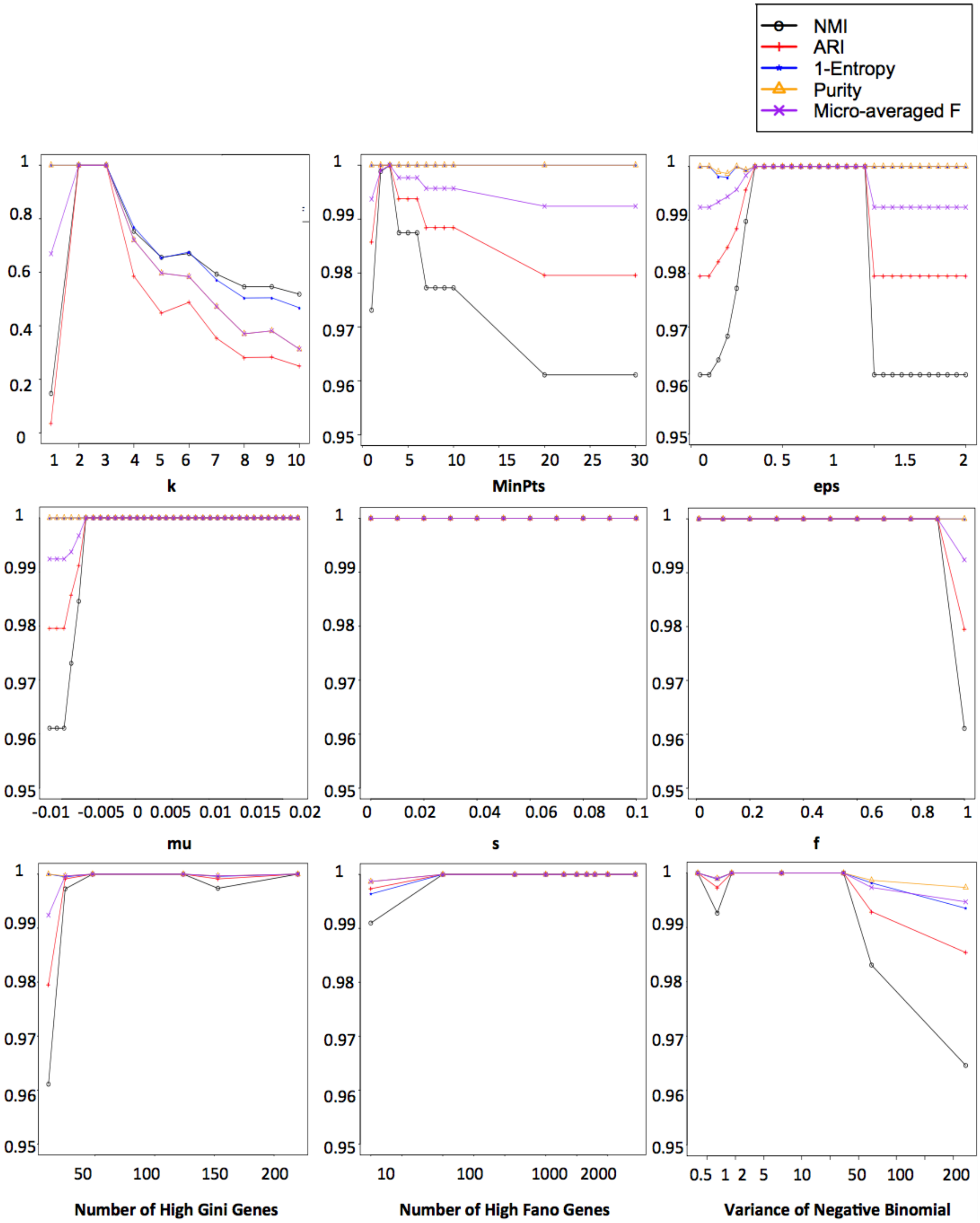
NMI
ARI
1-Entropy
Purity
Micro-averaged F
k
MinPts
eps
mu
s
f
Number of High Gini Genes
Number of High Fano Genes
Variance of Negative Binomial

Figure S9.

A sensitivity analysis for GiniClust2 on simulated data. Eight parameters were independently varied: k-means parameter k, DBSCAN parameters MinPts and eps, weighting scheme parameters μ, s, and f, and Gini and Fano gene thresholds. Additionally, the variance of the simulated data was varied. The effects of each of these changes on the final clustering accuracy of GiniClust2 were measured using several metrics: normalized mutual information (NMI), adjusted rand index (ARI), entropy, purity, and micro-averaged F-measure.

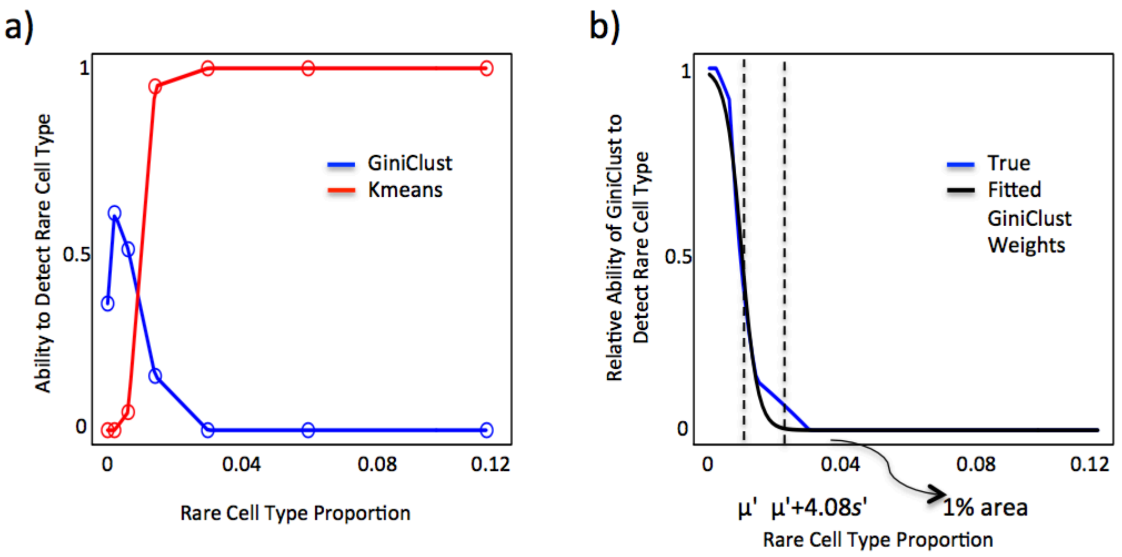

Figure S10. An evaluation of the abilities of GiniClust and Fano-factor-based k-means to detect rare cells, performed on the subsampled PBMC data sets. (a) Rare cell type detection abilities of GiniClust and Fano-factor-based k-means over a range of rare cell type proportions. (b) A representation of the ability of GiniClust to detect rare cell types over Fano-factor-based k-means, and its logistic fit. Parameters μ' and s' determine the shape of the curve.

2. Supplementary Table

Simulations	Macrophage	NK	B	Rare Cell Type Proportion
1-20.	5	1600	800	.002
21-40.	5	800	400	.004
41-60.	5	400	200	.008
61-80.	5	200	100	.016
81-100.	5	100	50	.032
101-120.	5	50	25	.063
121-140.	5	25	13	.116

Table S1. Cell numbers in three different cell types for each of 140 subsampled datasets from 68k PBMCs.
